# Long-Read Sequencing and *De Novo* Genome Assembly Pipeline of Two *Plasmodium falciparum* Clones (*Pf*3D7, *Pf*W2) Using Only the PromethION Sequencer from Oxford Nanopore Technologies without Whole-Genome Amplification

**DOI:** 10.3390/biology13020089

**Published:** 2024-01-31

**Authors:** Océane Delandre, Ombeline Lamer, Jean-Marie Loreau, Nasserdine Papa Mze, Isabelle Fonta, Joel Mosnier, Nicolas Gomez, Emilie Javelle, Bruno Pradines

**Affiliations:** 1 Unité Parasitologie et Entomologie, Département Microbiologie et Maladies Infectieuses, Institut de Recherche Biomédicale des Armées, 13005 Marseille, France; isabelle.fonta.09@gmail.com (I.F.); joelmosnier@orange.fr (J.M.); nico13dna@hotmail.com (N.G.); emilie.javelle@gmail.com (E.J.); bruno.pradines@gmail.com (B.P.); 2 Aix Marseille Univ, IRD, SSA, AP-HM, VITROME, 13005 Marseille, France; 3 IHU Méditerranée Infection, 13005 Marseille, France; 4 Unité Bactériologie, Département Microbiologie et Maladies Infectieuses, Institut de Recherche Biomédicale des Armées, 91220 Brétigny-sur-Orge, France; ombeline.lamer@def.gouv.fr; 5 Aix-Marseille Univ, INSERM, SSA, IRBA, MCT, 13005 Marseille, France; 6 French Armed Forces Center for Epidemiology and Public Health (CESPA), 13014 Marseille, France; jean-marie.loreau@def.gouv.fr; 7 Service de Biologie, Unité de Microbiologie, Hôpital Mignot, Centre Hospitalier de Versailles, 78150 Versailles, France; npapamze@gmail.com; 8 Centre National de Référence du Paludisme, 13005 Marseille, France

**Keywords:** genome assembly, *Plasmodium falciparum*, long-read sequencing, nanopore, PromethION, *Pf*3D7, *Pf*W2

## Abstract

**Simple Summary:**

This article proposes a biological and bioinformatic processing pipeline, from the sequencing library preparation to bioinformatics analysis, enabling the genome assembly (and without any amplification) of *Plasmodium falciparum*, the causal agent of malaria. All bioinformatic parameters are provided to enable everyone to use this pipeline.

**Abstract:**

Antimalarial drug resistance has become a real public health problem despite WHO measures. New sequencing technologies make it possible to investigate genomic variations associated with resistant phenotypes at the genome-wide scale. Based on the use of hemisynthetic nanopores, the PromethION technology from Oxford Nanopore Technologies can produce long-read sequences, in contrast to previous short-read technologies used as the gold standard to sequence Plasmodium. Two clones of *P. falciparum* (*Pf*3D7 and *Pf*W2) were sequenced in long-read using the PromethION sequencer from Oxford Nanopore Technologies without genomic amplification. This made it possible to create a processing analysis pipeline for human *Plasmodium* with ONT Fastq only. *De novo* assembly revealed N50 lengths of 18,488 kb and 17,502 kb for the *Pf*3D7 and *Pf*W2, respectively. The genome size was estimated at 23,235,407 base pairs for the *Pf3D7* clone and 21,712,038 base pairs for the *Pf*W2 clone. The average genome coverage depth was estimated at 787X and 653X for the *Pf*3D7 and *Pf*W2 clones, respectively. This study proposes an assembly processing pipeline for the human *Plasmodium* genome using software adapted to large ONT data and the high AT percentage of *Plasmodium*. This search provides all the parameters which were optimized for use with the software selected in the pipeline.

## 1. Introduction

Despite intensive efforts to eradicate malaria and the development of new combination therapies, it remains endemic in eighty-four countries. According to the 2023 report published by the World Health Organization (WHO), there were 248 million cases of malaria and 609,000 deaths in 2022 [1], compared to 229 million cases and 409,000 deaths in 2019. Since the 2000s, malaria cases have declined due to the development and use of rapid diagnostic tests, impregnated bed nets and new antimalarial drugs, including artemisinin-based combinations such as dihydroartemisinin–piperaquine, artemether–lumefantrine, and artesunate–amodiaquine [1]. However, *Plasmodium falciparum* has developed resistance to these new drugs, leading to therapeutic ineffectiveness and clinical failure, which constitutes a major public health problem [2,3]. The development of the genetic monitoring of parasite genomic variations is one of the most reliable approaches to assess susceptibility decreases and genetic polymorphisms [4].

The genomic knowledge of *Plasmodium falciparum* has evolved since the first genomic sequencing of the *Plasmodium falciparum* 3D7 clone took place in 2002 [5]. This clone was provided by limiting dilution from the NF54 isolate, recovered in the Netherlands from a malaria case airport, and is widely used in in vitro studies as a reference clone [6]. The whole-genome shotgun sequencing identified a genomic size of 22,853,764 base pairs coding for 5268 genes with 3465 hypothetical proteins. Fourteen chromosomes and two organelles were identified: the apicoplast and the mitochondria. The proportion of nucleotides (A + T) has been estimated at about 80.6% [7]. In 2019, the genome size was updated to 23,292,622 bp, with 5280 genes and 1776 hypothetical proteins [5]. By 2023, 5389 proteins had been annotated and 1626 proteins had been annotated as hypothetical proteins for a genome size around 23.33 Mb [8,9]. This sequenced genome is also the only reference genome used in genomics studies. *Pf*W2 was cloned from the Indochina III/CDC isolate, originally derived from a Laotian patient who failed chloroquine therapy [10], and no reference genomes are available for this clone. It is also used as reference clone in in vitro studies for its resistance to chloroquine.

Several sequencing tools have been developed, including Illumina sequencers (San Diego, CA, USA), that allow read sequencing between 150 and 300 base pairs [11]. Short-read sequencing with Illumina sequencers has become the reference for *Plasmodium falciparum* genomic studies [12]. However, short-read sequencing can be problematic for *de novo* assembly, due to the genome size and the many repeated regions. Pacbio sequencers have also been used for *Plasmodium* sequencing, but the gold standard remains illumina technology.

Nanopore technology was introduced in 2014 with the MinION sequencer [13]. This technology allows for long-read sequencing with a higher read depth when using the PromethION sequencer [11,13], and also enables high-throughput real-time analysis with a shorter processing time [14].

Since this implementation, however, few genomic studies have been published on *Plasmodium falciparum* due to the accessibility and price of sequencers (high cost) but also due to the richness of the genome in (A + T), which leads to a higher error rate [14]. These studies focus on nanopore sequencers, such as the MinION, to explore resistance in identified genes listed as causing antimalarial drug resistance [15,16,17,18].

In addition, most existing Plasmodium pipelines are suitable for Illumina short reads, like, for example, GATK4 [19], or pipelines are specialized in other, smaller microorganisms, and incompatible with Plasmodium genomics.

The aim of this study was to sequence, without any genomic amplification, *Plasmodium falciparum* clones 3D7 and W2 with the ONT technology with PromethION. The aim was then to *de novo* assemble their whole genomes using a Plasmodium-specific analysis pipeline. The aim was also to demonstrate the feasibility of nanopore whole-genome sequencing, considering the high AT richness of *Plasmodium*, which generally hinders sequencing.

## 2. Materials and Methods

### 2.1. Plasmodium falciparum Laboratory Cultures

The two clones (*Pf*3D7, *Pf*W2) used in this study were obtained from the Malaria Research and Reference Reagent Resource Center (MRA-102 and MRA-157) (BEI resources, Manassas, VA, USA).

The laboratory parasitic cultures were maintained in 4.5 mL of RPMI medium (Invitrogen, Paisley, UK) supplemented in 10% of human serum (EFS, Marseille, France) in 500 µL of human blood (A+, EFS, Marseille, France). Cultures were maintained under a controlled atmosphere: 37 °C, 5% CO_2_ and 10% O_2_. Cultures were maintained at high parasitaemia (>80% ring stage) to obtain an adequate amount of genetic material for DNA extraction. The RPMI medium was prepared with RPMI 1640 MEDIUM W/L-Glutamine and 26 mL of Hepes buffer (1M), 26 mL of sodium bicarbonate (7.5%), 3.2 mL of neomycin (10 mg/mL) and 1 mL of hypoxanthine (500 mg/L), orotic acid (250 mg/L) and L-Glutamine. Then, 20 mL of 10% D-glucose was added and, finally, was adjusted to a volume of 1 L with ultrapure water.

### 2.2. DNA Extraction

DNA from each sample was extracted using a QIAamp DNA blood Mini Kit (Qiagen, Hilden, Germany). Briefly, 200 µL of ATL buffer and 40 µL of proteinase k were mixed with 200 µL of each sample prior to a four-hour-long incubation at 56 °C, with agitation not exceeding 1200 rpm for sequencing. Each sample was supplemented with 200 µL of ethanol before adding wash buffer. DNA was eluted in 60 µL of ultrapure water and 1µL was quantified using the Qubit dsDNA high-sensitivity kit (Thermo Fisher Scientific, Waltham, MA, USA) following the manufacturer’s recommendation. DNA fragmentation was controlled with 9 µL of DNA on 0.7% agarose gel prepared with TBE 1x.

### 2.3. Whole-Genome Library

The whole-genome sequencing of *Plasmodium falciparum* clones was performed using the PromethION sequencer. After DNA extraction, without any amplification, a maximum of 1 µg of the DNA template was prepared using the ligation sequencing kit 110 (LSK110) (Oxford Nanopore technology, Oxford, UK) according to manufacturer’s instructions with the following minor modifications. The elution buffer was provided by Qiagen (Qiagen, Hilden, Germany) and the Short Fragment Buffer (SFB) from LSK 110 kit was used to keep all DNA fragments. The ratio of AMPure XP beads (Beckman Coulter, Brea, CA, USA) used for DNA purification was adapted at 1:1 for each step, where beads were used. The sequencing was performed for 24 h on R9.4.1 (FLO-PRO002) flow cells with sequencer default parameters and the “super high accuracy basecalling” was selected.

The sequencing time had to be adapted according to the sequencing library quality and the number of reads required at the end of sequencing.

### 2.4. Data Analysis

**Figure 1:**
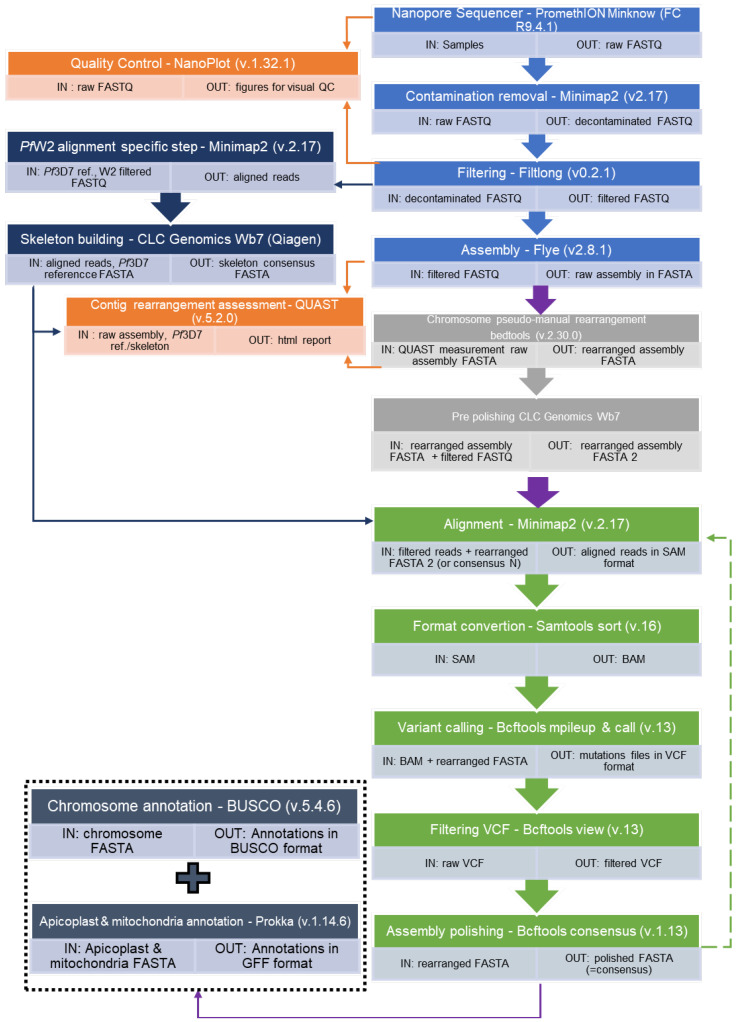
Assembly processing pipeline of *Plasmodium falciparum* Oxford Nanopore long-read sequencing. (Bash script is in Supplementary File S1). Raw assembly (Sky blue); optional, non automized assembly optimisation step (Grey); refinement processing (Green): the step should be processed as long as it reduces the reported mutation number (a repetition corresponds to following the green dotted arrow pathway). Quality control (Orange): to help set the parameters and thresholds. Optional skeleton build (Navy Blue): if no satisfying reference exists for your strain.

Bioinformatics analysis was performed on the fastq output data from the sequencer according to the pipeline presented in Figure 1 (bash script is in Supplementary File S1). Data quality control was verified using the NanoPlot software (v1.32.1) [20] and data were filtered using the Filtlong tool (v0.2.1) [21] to keep high-quality long reads only (https://github.com/rrwick/Filtlong, accessed on 10 January 2024). The Filtlong parameters were minimum length 5000, keep percent 90, and target bases 20,000,000,000 for *Pf*3D7, and minimum length 2000, keep percent 90, and target bases 20,000,000,000 for *Pf*W2. These parameters were adjusted according to the sequencing results. Parameters were set up to keep long reads.

#### 2.4.1. Genome Consensus of the *Plasmodium falciparum* 3D7 Clone

For the *Plasmodium falciparum* 3D7 clone, long reads were assembled using Flye (v2.8.1) [22], specifying the genome size as around 23 Mb and the “--asm-coverage” parameter as 35. The contig quality was checked using Quast (v5.0.2) [23] against the reference genome. Chromosomes were assembled manually using the *Plasmodium falciparum* 3D7 reference genome with the help of Bedtools (v2.30.0) [24] to polish the contigs. The apicoplast and the mitochondria were assembled in totality with Flye. Flye was chosen because it has had excellent results on various organisms and is versatile. Also, it was designed for assembly with error-prone reads and is based on k-mer and the graph theory [22,25].

#### 2.4.2. Genome Consensus of the *Plasmodium falciparum* W2 Clone

For the *Plasmodium falciparum* W2 clone, long reads were also assembled using Flye (v2.8.1) [22], specifying the genome size at around 23 Mb, but the “--asm-coverage” parameter was 300. This parameter was high to allow genome assembly. Since no reference genome was available for this clone, a consensus was created with Minimap2 (v.2.17) (“skeleton genome”) [26,27] (Figure 1).

The raw reads were mapped to the *Plasmodium falciparum* 3D7 reference genome, and the consensus was extracted using the CLC Genomics Workbench 7.5 (Qiagen) software. This process was performed to facilitate contig repair for chromosome assembly because of the high variability between the two clones. Chromosomes were assembled manually using the CLC consensus genome and Bedtools (v2.30.0) [24]. The apicoplast and the mitochondria were assembled in totality with Flye. This last step could be changed with the GreenHill software (v1.0.0) [28]. It should be noted that we had already repaired our genome using the technique described here, so we did not use the GreenHill software (v1.0.0).

#### 2.4.3. Final Consensus of the Two Clones

The assembly was refined for both clones. One refining event (also known as a “polishing step”) was associated with the following six sub-steps: firstly, minimap2 [26,27] was used to map filtered reads to the newly generated assembly, resulting in an SAM file. Secondly, the Samtools sort function was used to compress the SAM files into the BAM binary format. Thirdly, Bcftools mpileup and, fourthly, Bcftools call were used to identify all variants specifying the “--ploidy 1” parameter and to gather them inside a VCF file [29]. Bcftools view [29] was then used to filter the VCF file on the DP4 parameter with the filtering command: “((DP4[1] + DP4[2]) < (DP4[3] + DP4[4]) && (DP4[3] + DP4[4] > 40)”. Bcftools consensus was used to polish the assembly—that one assembly given to minimap2 in the first sub-step—from the filtered VCF file and to generate a new consensus assembly. To proceed to another refinement step, this consensus assembly could then be given to Minimap2 with the same filtered reads; otherwise, the refining process would be stopped at that point and the resulting consensus would be called the final consensus. That final consensus was, thus, a refined assembly assimilated to a complete genome sequence. This process was performed eleven times for *Pf*3D7 and twenty times for *Pf*W2.

#### 2.4.4. Genome Annotation

Genome annotations of both clones were performed using BUSCO (v5.4.6) and Metaeuk [30]. The data were uploaded to the Galaxy web platform, and we used the public server at usegalaxy.eu to analyse the data [31]. The BUSCO *Plasmodium* dataset includes 3642 genes from 23 species of *Plasmodium* [32]. BUSCO was run in eukaryotic mode.

A Venn diagram was created with BUSCO results and an R script. (All BUSCO data are provided in Supplementary Files S3–S5 and the R script is in the Supplementary File S6).

Whole-genome annotation was also performed with Companion (v2.2.4) for comparison with BUSCO [33].

#### 2.4.5. Apicoplast and Mitochondria Annotation

Gene prediction and protein annotation for the apicoplast and the mitochondria were performed using the Prokka software (v.1.14.6) [32,34]. The data were uploaded to the Galaxy web platform, and we used the public server at usegalaxy.eu to analyse the data [31].

### 2.5. Plasmodium falciparum 3D7 Reference Genome Variant Caller

The Filtlong-filtered Nanopore Fastq data were mapped to the *Pf*3D7 reference genome with the Minimap2 software [26,27]. The variant caller was run using bcftools mpileup (v1.13), bcftools call and bcftools view (v1.13) [29], specifying filter parameters on quality and on the DP4 parameter with the command “((DP4[1] + DP4[2]) < (DP4[3] + DP4[4]) && (DP4[3] + DP4[4] > 40)”. Bcftools stats were then used to create VCFs statistics, and the plot was generated with an R script (in Supplementary File S7).

The same pipeline was used to identify the difference between the *Pf*3D7 reference and the *Pf*W2 reads sequenced.

## 3. Results

### 3.1. Long-Read Sequencing Results

Long-read sequencing with PromethION initially produced reads with an estimated N50 length of 9731 kb for *Pf*3D7 and 16,305 kb for PfW2 (Table 1). The analyses were performed using NanoPlot from the raw fastq and the filtered fastq with Filtlong. Filtering made it possible to increase the quality score from 14.4 to 17 for the *Pf*3D7 clone and from 13.9 to 14.5 for the *Pf*W2 clone. For both clones, sequencing was performed for 24 h, resulting in over 12 million reads for *Pf*3D7 and over two million reads for *Pf*W2. After filtering, only 1,238,210 reads were retained for *Pf*3D7 and 1,296,307 for *Pf*W2. The assembly was thus carried out with reads having N50 lengths of 18,448 kb for the *Pf*3D7 clone and 17,502 kb for the *Pf*W2 clone (Table 1).

### 3.2. Plasmodium falciparum 3D7 De Novo Assembly

For *Pf*3D7, the assembly resulted in a total genome length of 23,477,924 base pairs distributed in 32 fragments. The average genome coverage depth was estimated to 787X by Flye (Table 2). Quast estimated the genome size to be 23,330,137 base pairs long, with 48 reported mis-assembly events on Flye out. The genome mapped with the *Pf*3D7 reference showed up to 99.94% similarity.

After *de novo* assembly, chromosomes were assembled manually from fragments using the *Pf*3D7 reference genome as a model and Bedtools. The chromosome quality was then assessed using Quast, ensuring errors were kept to a minimum. The last consensus sequence was obtained after multiple polishing steps involving VCF files. Ultimately, Quast estimated this genome size to 23,235,407 bp with 24 mis-assemblies, and the newly built genome fraction mapping with the *Pf*3D7 reference showed 99.348% similarity.

A final quality check was performed by mapping the filtered reads to both our new *Pf*3D7 genome and the older reference genome. This revealed that, although similar, the alignment rate was slightly lower than with the reference genome (94.20% identity compared to 94.18% identity, a difference which corresponds to 46,471 bp). This result is discussed in the discussion part.

### 3.3. Plasmodium falciparum W2 De Novo Assembly

The *Pf*W2 genome assembly resulted in a length of 23,302,768 base pairs distributed in 31 fragments and two scaffolds. The average genome coverage depth was estimated as 653X by Flye (Table 2). The Quast results on *Pf*W2 assembly showed that the differences between the two strains were too high to use the *Pf*3D7 reference genome. A “skeleton” genome was therefore created from the fastq file and the *Pf*3D7 reference genome, with the help of the CLC Genomics wb7 software. This step was essential to improve the genome. Chromosomes were assembled manually from fragments using the “skeleton” genome and Bedtools (method described in section number 2). Again, the last consensus sequence was obtained after multiple polishing steps involving the VCF file. The genome length was then estimated to be 21,712,038 bp long (Table 3). A final quality check was performed by mapping the filtered reads to our *PfW2* genome. It revealed that 99.14% reads mapped to the genome.

### 3.4. Genome Depth and Length

The consensus apicoplast and mitochondria for both genomes have similar lengths. However, *Pf*W2 chromosome lengths are still shorter than those of *Pf*3D7 (Table 3). Differences in genome size can be explained by the major deletions observed. This result is discussed in the Discussion part.

**Figure 2:**
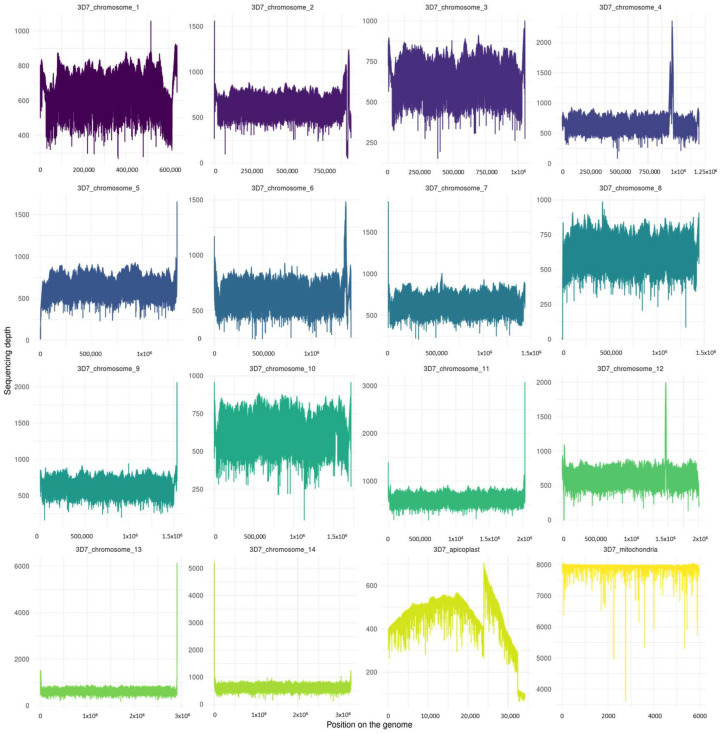
Sequencing depth of *Plasmodium falciparum* 3D7 clone genome assembly for the 14 chromosomes and the apicoplast and the mitochondria. The figure was realized with Samtools depth and R script (in Supplementary File S2). The mean depth per chromosomes were as follows: 3D7_chromosome_1 (754.4 ± 64.6); 3D7_chromosome_2 (764 ± 95.5); 3D7_chromosome_3 (768.3 ± 56.3); 3D7_chromosome_4 (808.9 ± 161.5); 3D7_chromosome_5 (774 ± 81.4); 3D7_chromosome_6 (784 ± 81.7); 3D7_chromosome_7 (779.7 ± 59.8); 3D7_chromosome_8 (762 ± 68.1); 3D7_chromosome_9 (765 ± 59.8); 3D7_chromosome_10 (760.8 ± 56.2); 3D7_chromosome_11 (769.6 ± 73.7); 3D7_chromosome_12 (774.8 ± 109.7); 3D7_chromosome_13 (779.7 ± 126); 3D7_chromosome_14 (776.9 ± 91.4); 3D7_apicoplaste (465.8 ± 113.4); and 3D7_mitochondria (7922.1 ± 151.9).

**Figure 3:**
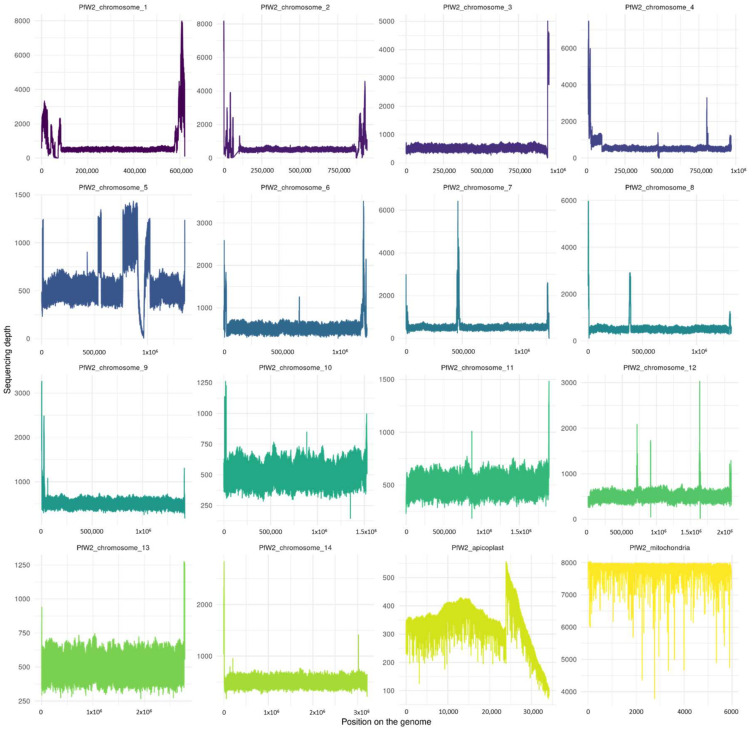
Sequencing depth of *Plasmodium falciparum* W2 clone genome assembly for the 14 chromosomes and the apicoplast and the mitochondria. The figure was realized with Samtools depth and R script (in Supplementary File S2). The mean depths per chromosome were as follows: PfW2_chromosome_1 (946 ± 1041.9); PfW2_chromosome_2 (731 ± 487.8); PfW2_chromosome_3 (669.7 ± 381); PfW2_chromosome_4 (749.4 ± 545.6); PfW2_chromosome_5 (705.9 ± 264.2); PfW2_chromosome_6 (669.7 ± 223.7); PfW2_chromosome_7 (685.7± 366.4); PfW2_chromosome_8 (660.6 ± 334.2); PfW2_chromosome_9 (632.8 ± 144.6); PfW2_chromosome_10 (627.5 ± 62.8); PfW2_chromosome_11 (620.9 ± 59.5); PfW2_chromosome_12 (635.9 ± 123.6); PfW2_chromosome_13 (624.8 ± 58.8); PfW2_chromosome_14 (630.5 ± 98.5); PfW2_apicoplaste (348.2 ± 80.6); and PfW2_mitochondria (7823.6 ± 251.4).

The depth per chromosome was measured for each base pair of both consensus genomes (Figure 2 and Figure 3). The most covered region of each clone was their mitochondria, with a depth nearing 7900X.

### 3.5. Genome Annotation

**Figure 4:**
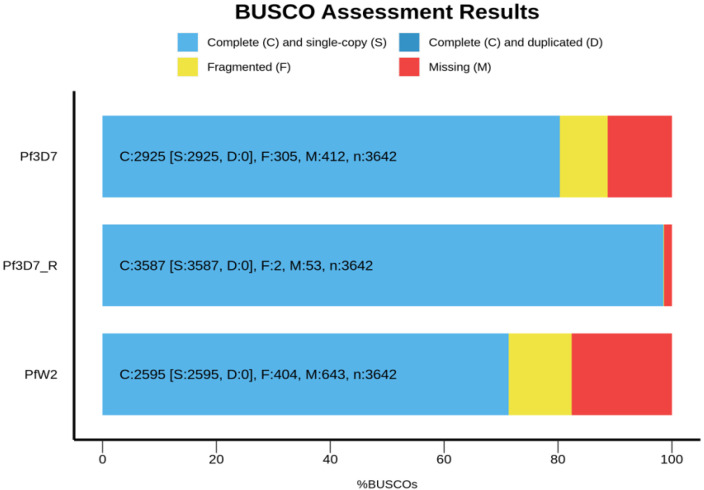
BUSCO completeness results. *Pf*3D7, *Pf*W2 consensus genomes and the *Pf*3D7 reference genome were used. The three genomes were annotated with BUSCO and Metaeuk on Galaxy platform [31]. The figure was realized with the BUSCO.py script [32].

For the assembled *Pf*3D7 consensus sequence, BUSCO identified 2925 complete genes out of the 3642 in the database. A total of 305 genes were fragmented and 412 were missing (Figure 3). In comparison, for the *Pf*3D7 reference genome, BUSCO identified 3587 genes out of the 3642, including 2 fragmented genes and 53 missing genes (Figure 4).

For the *Pf*W2 consensus sequence, 2595 complete genes were identified out of the 3642 in its database: 404 genes were fragmented and 643 were missing (Figure 4).

**Figure 5:**
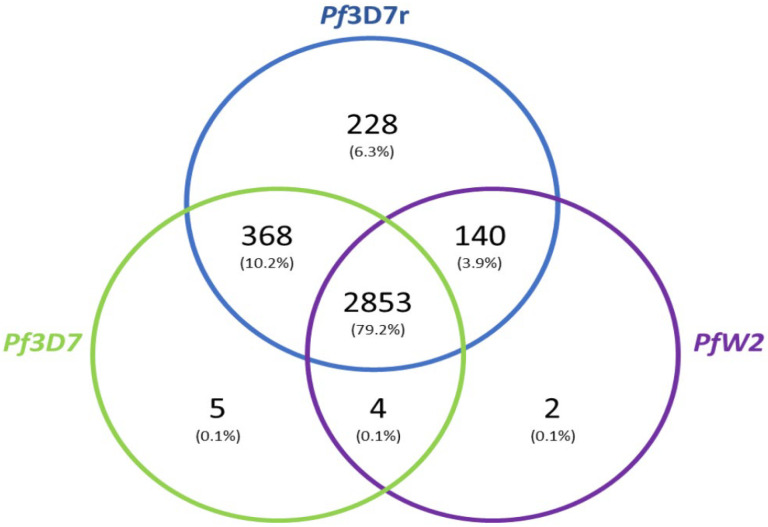
Venn diagram showing the number distribution of shared genes between the three *Plasmodium falciparum* clones. The Venn diagram shows the genes shared by the three strains, whether fragmented or complete. Based exclusively on BUSCO data. The *Pf*3D7r is the reference genome. Missing genes were not represented. (R script is in Supplementary File S6).

The BUSCO results were compared with one another to identify which genes were common to the clones and the *Pf*3D7 reference. The Venn diagram shows that 2853 genes are shared between the three clones and 228 genes are present only in the reference. Five genes are present only in the *Pf*3D7 sequenced and two genes are present only in the *Pf*W2 sequenced (in chromosome 3). A total of 368 genes are shared only by the *Pf3D7* clone and the *Pf*3D7 reference and 140 genes are shared only by the *Pf*3D7 reference and the *Pf*W2 (Figure 5). All BUSCO data are presented in Supplementary Files S3–S5.

The Companion software (v2.2.4) was also used to compared BUSCO annotation. For *Pf3D7*, 3477 genes were identified out of 5562 genes of the database, and for *Pf*W2 this was 1567 genes out of 5562 of the databases. For both, the *P. falciparum* 3D7 database reference was used. A total of 2535 and 4060 pseudogenes were identified for *Pf*3D7 and *Pf*W2, respectively. This result is discussed in the Discussion part.

### 3.6. Apicoplast and Mitochondria Annotations

The apicoplast and mitochondria were annotated using the Prokka software in Galaxy. Prokka was chosen because it is a software specializing in the annotation of prokaryotic genomes and can manage circular genomes. The two organelles are highly similar to prokaryote genomes.

Thirty CDS, thirty tRNA and four rRNA were identified for the newly assembled *Pf3D7* apicoplast. As for *Pf*W2, 29 CDS, 33 tRNA and 4 rRNA were identified.

Three CDS were identified within the mitochondria. These CDS are shared by the three studied *Plasmodium*.

### 3.7. Genomic Variability of the Pf3D7 Clone against the Pf3D7 Reference Genome

**Figure 6:**
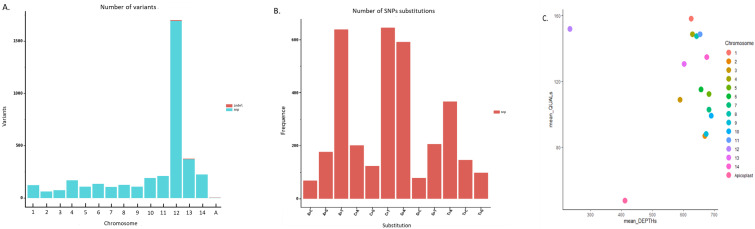
Variability between the *Pf3D7* clone sequencing and the *Pf3D7* reference genome. (**A**) Variants observed chromosome by chromosome. The plot was generated with the filtered VCF file (R script is in Supplementary File S7). Each number (1–14) corresponds to a chromosome (1–14) and “A” to the apicoplast. No variant was observed in the mitochondria. (**B**) Number of SNP substitution for whole genome of *Pf3D7* genome reference. (**C**) Mean quality versus mean depths for the variants observed.

The sequenced *Pf*3D7 clone reads were mapped to the reference genome and revealed a very high variability throughout the genome. The variant call format revealed 3719 variants between the reference *Pf*3D7 genome and the *Pf*3D7 sequenced reads (Figure 6A), and most variations are in chromosome 12. In addition, the most observed substitutions were A > T, C > T and G > A (Figure 6B). For the observed variants, quality was compared with the sequencing depth. Chromosome 12 had a good average quality but low depth. As for the apicoplast, both parameters were low. Other chromosomes shared a high mean depth (between 600X and 700X) and a high average quality (≥80) (Figure 6C).

No mutations were observed inside the mitochondria.

### 3.8. Genomic Variability of the PfW2 Clone against the Pf3D7 Reference Genome

The *Pf*W2 reads sequenced were mapped onto the *Pf*3D7 reference genome and after a variant call, 100,000 variants were identified between them, which represent 0.43%.

## 4. Discussion

In this study, a bioinformatics assembly pipeline was developed for the human parasite *Plasmodium falciparum* using only nanopore long-read sequencing from library preparation to *de novo* genome assembly. The selected software packages were chosen for their compatibility with Nanopore fastq and their ability to analyse *Plasmodium falciparum* data. Each software parameter is specified for more practical use.

This pipeline enabled the *de novo* assembly of two whole genomes with a substantial sequencing depth never archived before. A high sequencing depth is necessary to compensate for the sequencing errors of nanopore technology. However, the errors generated during sequencing cannot be ignored and are mainly due to the high AT content (80.6%) of *Plasmodium* and its sequenced genome size (23 Mb) [7]. In addition, in this study the LSK110 ligation kit was used to prepare the sequencing library and Oxford Nanopore Technologies now recommends using the LSK114 kit ligation chemistry (Oxford Nanopore Technology, Oxfrod, UK), which would create even fewer sequencing errors. We were also confronted with the bioinformatic limitations of genome repair. Some base pairs in the consensus genome did not match the sequenced reads, despite the many polishing steps performed to generate the consensus. These sequencing errors were highlighted with the VCF files and are in the order of 0.0004% over the whole genome, which remains very low. We shall provide some insight about its potential cause in our discussion of bioinformatics issues. Finally, these errors are counterbalanced by the sequencing depth obtained for the two strains sequenced here, enabling the creation of a robust genome.

One of the main limitations of Oxford Nanopore Technologies is the quantity of DNA required. ONT recommends loading 1 µg of DNA into PromethION Flow Cells. For clinical samples, this protocol therefore needs to be adapted by using filtration columns to remove human DNA according to the protocol presented by Coppée et al. [35], followed by whole-genome amplification for small parasitaemia [36]. This protocol was used to sequence the Plasmodium clinical sample by nanopore adaptive sampling, which shows that the technology can be adapted to clinical isolates with very low parasitaemia [37].

With regard to bioinformatics assembly, high-peak and low-depth regions (Figure 1 and Figure 2) at the start and end of chromosome assemblies correspond, respectively, to an underestimation or overestimation of the repeated-region repeat number. It is a known pattern associated with the presence of telomeres. Such a result was, therefore, expected. Other patterns of sudden increases and falls in depth alongside a chromosome may correspond to a mis-assembly pattern due to an incorrect number of repeats for repeated regions. This might be especially true if the depth of this specific region is a multiple of the surrounding region depth.

We can observe that the *Pf*3D7 and *Pf*W2 clones both have regions that have a higher depth of coverage than others. This is particularly visible in the apicoplast. This observation could result from a repeated region with a length or number of repeats which was underestimated by Flye during the assembly. Indeed, the resolution of extensively large, repeated regions is one of the major troubleshootings of *de novo* assembly. Despite long-read sequencing technology being devised to bypass repeated regions, some regions are still too wide for all repetitions to be encompassed within long reads.

As mentioned above, assemblies were carried out with N50 lengths of 18,448 kb and 17,502 kb for each of the *Pf*3D7 and *Pf*W2 clones. In *Pf*3D7, the higher-depth apicoplast region corresponded approximately to the 23,750–31,250 bp segment, hence a 7500 base-long segment. This is supposedly shorter than the N50 length, so the assembly should have been resolved. However, as the depth in this particular segment was twice as high as the other surrounding regions within the apicoplast, it is most likely that the segment should have been a two-time repeated segment. Hence, the length ranged from 7500 to 15,000 base pairs long, nearing the N50 length. If the assembly software was lacking reads for the resolution of this particular region—which seems to be the case at the end of the segment, considering the far-below-average depth (see Figure 2)—it could have opted for an inappropriate alternative assembly, although algorithmically correct assembly.

This type of reasoning can be applied to other regions within the genome. A repeated-region repeat underestimation is also most likely what happened for the ninth chromosome of clone *Pf*W2, as some regions have a near-stagnant depth of 1000X while the majority of chromosomes have a depth of 500X. Hence, some regions should have been duplicated (rather than truncated during the assembly). In contrast, another region is far less represented. As it is enclosed between two underestimated regions (i.e., these regions are missing a copy inside the final assembly), it is most likely that this region was present only in one of the supposed duplicates.

In addition, we can observe that some peaks are very high in other chromosomes. This could indicate a small specific segment in which the number of repeats was greatly underestimated during assembly.

Inside the VCF file, and despite the numerous polishing steps of the assembled genomes, assembly errors were persistent. This could be explained by the underestimated repetition number of repeated regions. Indeed, for a repeated region that should have been duplicated (but has only one copy inside the assembly), the mapped reads would report around 50% of the current base and around 50% of another base, which would then be written inside the VCF file for the next polishing step. Each subsequent polishing step would then only correspond to a switch between the other of the two bases. This would continue indefinitely.

Despite these mis-assembly events, the produced *de novo* assemblies are still of use. They present at least one copy of the underestimated regions, thus enabling read mapping. Indeed, the alignment of the trimmed reads to the final assembly reached 94.20% for *Pf*3D7 and 99.14% for *Pf*W2. It could thus be used to compare genomes in reference to these novel assemblies and more modern strains. It may also support exploratory RNA-seq studies and other omics studies.

Unfortunately, we did not use the ILRA pipeline to improve long-read assemblies because the research was published in July 2023 and we assembled our genomes before the publication [38]. Manual repair steps can be replaced by the ILRA pipeline. To develop their pipelines, Ruiz et al. used Plasmodium reads sequenced with PacBio technology, and also used two fungal genomes sequenced with ONT technology.

Finally, in this study, the genomic variability of *Plasmodium falciparum* clone 3D7 was highlighted. This variability may be the result of culture adaptation, which would potentially explain why BUSCO did not identify all the genes. However, it could also be due to a lack of data inside the BUSCO database. A comparison between BUSCO and Companion was also carried out in order to identify the best software for annotation. Both software packages showed limitations in annotation, which could indicate too much in vitro genomic variability. In fact, many stop codons were found by visualising the genome using IGV software (v11.03.13). According to Claessens et al. [39], in vitro variability has already been observed. Thus, it would most likely be due to laboratory culture adaptation rather than that of an insufficient BUSCO or Companion database. Comparison of the two clones revealed a difference of 100,000 base pairs. This difference can be explained by culture adaptability, and also by sequencing errors due to the technology employed as well as genetic drift of the clone.

In future, this pipeline could also produce a hybrid genome with the addition of short reads, produced for example, by Illumina. Using both techniques would improve the robustness of the sequenced genome in a complementary way. Hybrid genome assembly can be useful for clinical isolates presenting therapeutic failures unexplained by currently known molecular markers. Nanopore technology also makes it possible to study DNA methylation profiles, which would enable the study of resistances that are epigenetic rather than genetic [40].

## 5. Conclusions

This study proposes an assembly processing pipeline from the biological to bioinformatic analysis for the human *Plasmodium* genome. It enables the assembly of complex *Plasmodium* genomes (80.6% AT) using exclusively ONT long reads without any genomic amplification. This pipeline is useful for the analysis of clinical isolates, but first filtration would be necessary in a laboratory.

However, it might still need to be optimized and adapted to each clinical isolate, meaning that the software parameters and thresholds should be overhauled. In the perspective of this work, we could make a hybrid genome composed of Illumina and ONT reads.

## Figures and Tables

**Table 1:** NanoPlot data for the *Pf*3D7 and *Pf*W2 clones. NanoPlot analyses were performed on the fastq files from the sequencer output and from the fastq files filtered by the Filtlong software (filter parameters are in the method section). (*Pf*3D7: *Plasmodium falciparum* 3D7 clone; *Pf*W2: *Plasmodium falciparum* W2 clone; * data are in kb).

Nanoplot Data	*Pf*3D7 Reads	*Pf*3D7 Filtered Reads	*Pf*W2 Reads	*Pf*W2 Filtered Reads
Mean read length *	3440.2	15,843.6	7701.3	11,600.2
Mean read quality	14.4	17.0	13.9	14.5
Median read length *	1037.0	12,363.0	4068.0	7903.0
Median read quality	14.1	17.1	14.0	14.6
Number of reads	12,854,191.0	1,238,210.0	2,192,635.0	1,296,307.0
Read length N50 *	9731	18,448	16,305	17,502
Total bases	44,221,405,060.0	19,617,651,842.0	16,886,156,110.0	15,037,370,421.0

**Table 2:** *De novo* assembly data for *Pf*3D7 and *Pf*W2 clones. *De novo* assembly was performed using the Flye software (all parameters are in the method section).

	*Pf*3D7	*Pf*W2
Total length	23,477,924	23,302,768
Fragments	32	31
Fragments N50	1,265,374	1,700,513
Largest fragments	3,284,512	3,249,547
Scaffolds	0	2
Mean coverage	787	653
N_50_ (Kb)	18,488	17,502
N_90_	8461	5277

**Table 3:** Chromosome length and average assembly depth for *Pf*3D7 and *Pf*W2.

	*Pf*3D7		*Pf*W2	
	Length	Average Depth	Length	Average Depth
chromosome 1	638,193	754.4 ± 64.6	621,378	946 ± 1041.9
chromosome 2	940,408	764 ± 95.5	942,789	731 ± 487.8
chromosome 3	1,056,079	768.3 ± 56.3	955,041	669.7 ± 381
chromosome 4	1,195,288	808.9 ± 161.5	969,939	749.4 ± 545.6
chromosome 5	1,338,524	774 ± 81.4	1,353,934	705.9 ± 264.2
chromosome 6	1,412,742	784 ± 81.7	1,294,864	669.7 ± 223.7
chromosome 7	1,438,736	779.7 ± 59.8	1,272,765	685.7± 366.4
chromosome 8	1,445,520	762 ± 68.1	1,320,440	660.6 ± 334.2
chromosome 9	1,534,997	765 ± 59.8	1,420,162	632.8 ± 144.6
chromosome 10	1,716,863	760.8 ± 56.2	1,528,816	627.5 ± 62.8
chromosome 11	2,029,548	769.6 ± 73.7	1,894,263	620.9 ± 59.5
chromosome 12	2,257,511	774.8 ± 109.7	2,097,388	635.9 ± 123.6
chromosome 13	2,913,737	779.7 ± 126	2,786,778	624.8 ± 58.8
chromosome 14	3,277,058	776.9 ± 91.4	3,213,288	630.5 ± 98.5
apicoplast	34,237	465.8 ± 113.4	34,226	348.2 ± 80.6
mitochondria	5966	7922.1 ± 151.9	5967	7823.6 ± 251.4
total length	23,235,407		21,712,038	

## Data Availability

This project was registered under the BioProject accession number PRJNA987860, and the SAMN36493565 (*Pf*3D7) and SAMN36493566 (*Pf*W2) Biosample accession numbers. The genomes have been deposited in NCBI (987860[BioProject]—Nucleotide—NCBI (nih.gov) (accessed on 29 January 2024). Sequencing report, BUSCO annotation results, R scripts, and bash script used in the pipeline are in the Supplementary Files.

## Supplementary Materials

The following supporting information can be downloaded at: https://www.mdpi.com/article/10.3390/biology13020089/s1, Supplementary File S1: Bash script assembly pipeline. This script provides the command lines that can be used in an Ubuntu terminal and the parameters used for each software. Supplementary File S2: R script for chromosome depth coverage. This R script was used to produce Figure 2 and Figure 3 at chromosome depth. Supplementary File S3: BUSCO annotation results for *Pf*3D7 assembly. The results were obtained from the Galaxy platform with the *Pf*3D7 consensus assembly. Supplementary File 4: BUSCO annotation result for *PfW2* assembly. The results were obtained from the Galaxy platform with the *Pf*W2 consensus assembly. Supplementary File S5: BUSCO annotation result for *Pf*3D7 reference genome. The results were obtained from the Galaxy platform with the *Pf*3D7 reference genome. Supplementary File S6: R script for BUSCO comparison and Ven diagram creation. This R script was used to produce Figure 4 by comparing BUSCO data obtained for the three genomes. Supplementary File S7: R script for VCF analysis. This script was used to produce Figure 6.
